# Exploratory factor analysis with structured residuals for brain network data

**DOI:** 10.1162/netn_a_00162

**Published:** 2021-02-01

**Authors:** Erik-Jan van Kesteren, Rogier A. Kievit

**Affiliations:** Utrecht University, Department of Methodology and Statistics, Utrecht, the Netherlands; University of Cambridge, MRC Cognition and Brain Sciences Unit, Cambridge, UK

**Keywords:** Dimension reduction, Exploratory Factor analysis, Structural covariance, Functional connectivity, Symmetry, Structural equation model

## Abstract

Dimension reduction is widely used and often necessary to make network analyses and their interpretation tractable by reducing high-dimensional data to a small number of underlying variables. Techniques such as exploratory factor analysis (EFA) are used by neuroscientists to reduce measurements from a large number of brain regions to a tractable number of factors. However, dimension reduction often ignores relevant a priori knowledge about the structure of the data. For example, it is well established that the brain is highly symmetric. In this paper, we (a) show the adverse consequences of ignoring a priori structure in factor analysis, (b) propose a technique to accommodate structure in EFA by using structured residuals (EFAST), and (c) apply this technique to three large and varied brain-imaging network datasets, demonstrating the superior fit and interpretability of our approach. We provide an R software package to enable researchers to apply EFAST to other suitable datasets.

## INTRODUCTION

Using modern imaging techniques, it is possible to investigate brain networks involving many regions, across different modalities such as grey matter volume, white matter tracts, and functional connectivity. To examine the relation of these networks with external variables of interest, it is often necessary to summarize them, using a small number of dimensions, often called factors or components. These low-dimensional components representing the networks can be tracked over the lifespan (de Mooij, Henson, Waldorp, & Kievit, 2018; DuPre & Spreng, 2017), compared to behavioural measures (Colibazzi et al., 2008), or related to phenotypes such as intelligence (Ferguson, Anderson, & Spreng, 2017). In the fields of statistics and mathematics, such methods for making analyses tractable and interpretable are collectively called dimension reduction.

Many popular dimension reduction techniques make use of *covariance*. For example, principal components analysis (PCA) can be estimated using only a decomposition of the covariance matrix. Covariance underlies many brain imaging and network analysis approaches, too: in analysis of structural connectivity, regions of grey matter volume or white matter tractography which covary across individuals may constitute connected networks (Alexander-Bloch, Giedd, & Bullmore, 2013; Mechelli, Friston, Frackowiak, & Price, 2005), and in resting-state fMRI analysis, regions which covary within an individual over time are considered to have a functional connection (Van Den Heuvel & Pol, 2010). Thus, dimension reduction on the basis of covariance matrices is directly applicable to the field of network neuroscience.

Exploratory factor analysis (EFA) is one such method for dimension reduction based on covariance. EFA models the observed covariance matrix of a set of *P* variables by assuming there are *M* < *P* factors, which predict the values on the observed variables. Although other techniques such as PCA and independent component analysis (ICA) are more common in neuroimaging analysis, EFA has been used since the early days of MRI (see McIntosh & Protzner, 2012, for a review and Machado, Gee, & Campos, 2004 for an early methodological investigation). For instance, Tien et al. (1996) performed an EFA on 60 controls and 44 schizophrenia patients for a selection of regions of interest, explicitly noting the high degree of left/right symmetry and a disruption of this symmetry in patients. Similarly early studies used EFA to model morphology (Stievenart et al., 1997) and width (Denenberg, Kertesz, & Cowell, 1991) of the corpus callosum. Some approaches combined structural equation modeling (SEM) and PCA to model latent factors of grey matter structure in clinical populations (Yeh et al., 2010). These approaches have also been used to study typical population of children and adults (Colibazzi et al., 2008). More recently, EFA has been used to reduce individual differences in white matter microstructure in clinical populations (Herbert et al., 2018), as well as (extremely) large-scale population studies (Cox et al., 2016). Hybrid approaches have combined exploratory and confirmatory factor analysis approaches (Baskin-Sommers, Neumann, Cope, & Kiehl, 2016; de Mooij et al., 2018) and used EFA in multimodal structural acquisitions (Mancini et al., 2016). EFA has also been used for functional imaging, including both fMRI (e.g., James et al., 2009) and EEG (Scharf & Nestler, 2018; Tucker & Roth, 1984). Most excitingly, recent work has used EFA to compare and contrast patterns of individual differences in brain structure at baseline with individual differences in developmental change over time, noting striking differences in dimensionality of change versus cross-sectional differences (Cox et al., 2020). Although the above is not intended to be a comprehensive review, it shows that EFA has been used widely in the imaging literature since early days.

Many related dimension reduction techniques exist beyond EFA, including partial least squares (PLS), ICA, spectral decomposition, and many more beyond our current scope (see Roweis & Ghahramani, 1999; Sorzano, Vargas, & Montano, 2014). All of these techniques aim to approximate the observed data by means of a lower dimensional representation. These techniques, although powerful, share a particular limitation, at least in their canonical implementations, namely that they cannot easily integrate prior knowledge of (additional) covariance structure present in the data. In other words, all observed covariation is modeled by the underlying factor structure.

This limitation is relevant in the context of structural and functional brain connectivity data because of *symmetry*: Much like other body parts, contralateral (left/right) brain regions are highly correlated due to developmental and genetic mechanisms which govern the gross morphology of the brain. Ignoring this prior information will adversely affect the dimension reduction step, leading to worse representation of the high-dimensional data by the extracted factors. Simple workarounds, such as averaging left and right into a single index per region, have other drawbacks: they throw away information, preclude the discovery of (predominantly) lateralized factors, and prevent the study of (a)symmetry as a topic of interest in and of itself.

Other classes of techniques, developed largely within psychometrics, can naturally accommodate additional covariance structure such as symmetry. These techniques started with multitrait-multimethod (MTMM) matrices (Campbell & Fiske, 1959) and later confirmatory factor analysis (CFA) with residual covariances (e.g., Kenny, 1976). MTMM is designed to extract factors when these factors are measured in different ways: when measuring personality through a self-report questionnaire and behaviour ratings, there are factors that explain correlation among items corresponding to a specific trait such as “extraversion,” and there are factors that explain additional correlation between items because they are gathered using the same methods (self-report and behavioural ratings). Thus MTMM techniques separate the correlation matrix into two distinct, summative parts: correlation due to the underlying traits (factors) of central interest, and correlation due residual structure in the measurements. However, MTMM requires a priori knowledge of the trait structure (e.g., the OCEAN model of personality) for estimation.

In this paper, we combine dimension reduction (e.g., across many brain regions) and prior structure knowledge (e.g., symmetry) by introducing EFA with structured residuals (EFAST). EFAST builds on standard implementations of EFA, CFA, and MTMM, but goes beyond these techniques by simultaneously allowing for exploration and the incorporation of residual structure. We show that EFAST outperforms EFA in empirically plausible scenarios, and that ignoring the problem of structured residuals in these scenarios adversely affects inferences.

This paper is structured as follows. First, we explain why using standard EFA or CFA for brain imaging data may lead to undesirable results, and we develop EFAST based on novel techniques from SEM. Then, we show that EFAST performs well in simulations, demonstrating superior performance compared to EFA in terms of factor recovery, factor covariance estimation, and the number of extracted factors when dealing with symmetry. Third, we illustrate EFAST in a large neuroimaging cohort (Cam-CAN; Shafto et al., 2014). We illustrate EFAST for three distinct datasets: grey matter volume, white matter microstructure, and within-subject fMRI functional connectivity. We show how EFAST outperforms EFA both conceptually and statistically in all three datasets, showing the generality of our technique. We conclude with an overview and suggestions for further research.

Accompanying this paper, we provide tools for researchers to use and expand upon with their own datasets. These tools take the form of (a) an R package called EFAST and a tutorial with example code (van Kesteren & Kievit, 2020a), and (b) synthetic data and code to reproduce the empirical examples and simulations (van Kesteren & Kievit, 2020b).

## FACTOR ANALYSIS WITH STRUCTURED RESIDUALS

In this section, we compare and contrast existing approaches in their ability to perform factor analysis in an exploratory way while at the same time accounting for residual structure. We discuss new developments in the field of exploratory structural equation modeling (ESEM) that enable simultaneous estimation of exploratory factors and structured residuals, after which we develop the EFAST model as an ESEM with a single exploratory block. We will use brain morphology data with bilateral symmetry as our working example throughout, although the principles here can be generalized to datasets with similar properties.

EFA, as implemented in software programs such as SPSS, R, and Mplus, models the observed correlation matrix through two summative components: the factor loading matrix **Λ**, relating the predefined number *M* of factors to the observed variables, and a diagonal residual variance matrix **Θ**, signifying the variance in the observed variables unexplained by the factors. Using maximum likelihood, principal axis factoring, or least squares (Harman & Jones, 1966), the factor loadings and residual variances are estimated such that the implied correlation matrix **Σ** = **ΛΛ**^*T*^ + **Θ** best approximates the observed correlation matrix ***S***. After estimation, the factor loadings are rotated to their final interpretable solution by using objectives such as oblimin, varimax, or geomin (Bernaards & Jennrich, 2005).

**Figure 1.**
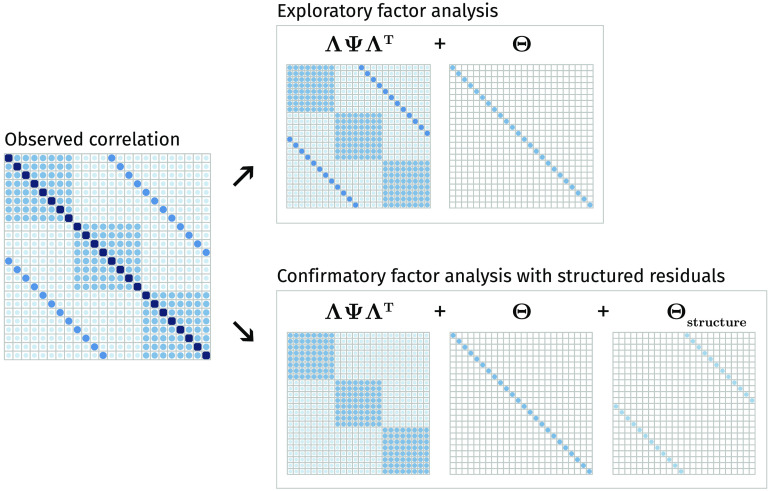
Example observed correlation matrix and its associated decomposition according to EFA (top) and according to CFA (bottom) into a factor-implied correlation component (**ΛΨΛ**^*T*^), residual variance component **Θ**, and—in CFA with residual structure only—residual structure component.

We illustrate the challenge and the rationale behind our approach in Figure 1. The true correlation matrix is highlighted on the left, with correlations due to three factors shown as diagonal blocks. However, there is also considerable off-diagonal structure: the secondary diagonals show a symmetry pattern similar to that observed in real-world brain structure data (Taylor et al., 2017). The top panel of Figure 1 shows that a traditional EFA approach will separate this data matrix into two components: (a) covariance due to the hypothesized factor structure and (b) the diagonal residual matrix. The key challenge is that EFA will attempt to approximate all the off-diagonal elements of the correlation matrix through the factors, even if this adversely affects the recovery of the true factor structure. Performing EFA with such a symmetry pattern may affect the factor solution in a variety of ways. For instance, in this toy example, the EFA model requires more than 12 factors to represent the data, instead of the 3 factors specified (see Supporting Information Figure S1). In other words, in such cases it is essential to incorporate the known residual structure via a set of additional assumptions.

As an alternative to EFA, we may implement a CFA instead. In contrast to EFA, CFA imposes a priori constraints on the **Λ** matrix: some observed variables do not load on some factors. Moreover, in contrast to standard EFA approaches, residual structure can be easily implemented in CFA by using standard SEM software such as lavaan (Rosseel, 2012). In other words, CFA would allow us to tackle the problem in Figure 1: We can allow for the residual structure known a priori to be present in the data. By allowing for the residual structure in the data, a CFA yields the implied matrices shown in the bottom panel of Figure 1, retrieving the correct factor loadings, residual variance, and residual structure. However, this is only possible because in this toy example we *know* the factor structure. In many empirical situations this is precisely what we wish to discover. In the absence of theory about the underlying factors, it is thus not possible to benefit from these features of CFA.

As such, we need an approach that can combine the strengths of EFA (estimating the factor structure in the absence of strong a priori theory) with those from CFA (the potential to allow for a priori residual structure). Here, we propose a hybrid between the two, which we call exploratory factor analysis with structured residuals, or EFAST. In order to implement and estimate these models, we make use of recent developments in the field of SEM. In the next section, we explain how these developments make EFAST estimation possible.

### Exploratory SEM

Exploratory SEM (ESEM) is an extension to SEM which allows for blocks of exploratory factor analysis within the framework of confirmatory SEM (Asparouhov & Muthén, 2009; Brown, 2006; Guàrdia-Olmos, Peró-Cebollero, Benítez-Borrego, & Fox, 2009; Jöreskog, 1969; Marsh, Morin, Parker, & Kaur, 2014; Rosseel, 2019). ESEM is a two-step procedure. In the first step, a regular SEM model is estimated, where each of the EFA blocks have a diagonal latent covariance matrix **Ψ** and the **Λ** matrix of each block is of transposed echelon form, meaning all elements above the diagonal are constrained to 0. For a nine-variable, three-factor EFA block *b* the matrices would then be:

$$\Psi_{b}=\left[\begin{array}{ccc} 1 & 0 & 0\\ 0 & 1 & 0\\ 0 & 0 & 1 \end{array}\right],\;\Lambda_{b}=\left[\begin{array}{ccc} \lambda_{11} & 0 & 0\\ \lambda_{21} & \lambda_{22} & 0\\ \lambda_{31} & \lambda_{32} & \lambda_{33}\\ \lambda_{41} & \lambda_{42} & \lambda_{43}\\ \lambda_{51} & \lambda_{52} & \lambda_{53}\\ \lambda_{61} & \lambda_{62} & \lambda_{63}\\ \lambda_{71} & \lambda_{72} & \lambda_{73}\\ \lambda_{81} & \lambda_{82} & \lambda_{83}\\ \lambda_{91} & \lambda_{92} & \lambda_{93} \end{array}\right]$$

This means there are $M_{b}^{2}$ constraints for each EFA block *b*. This is the same number of constraints as conventional EFA (Asparouhov & Muthén, 2009). The second step in ESEM is to rotate the solution by using a rotation matrix ***H***. Just as in regular EFA, this rotation matrix is constructed using objectives such as geomin or oblimin. In ESEM, the rotation affects the factor loadings and latent covariances of the EFA blocks, but also almost all other parameters in the model (Asparouhov & Muthén, 2009) provide an overview of how rotation changes these parameter estimates). Despite these changes, a key property of ESEM is that different rotation solutions lead to the same overall model fit.

ESEM has long been available only in Mplus (Asparouhov & Muthén, 2009; Muthén & Muthén, 1998). More recently, it has become available in open sourced R packages psych (for specific models, Revelle, 2018) as well as lavaan (since version 0.6-4, Rosseel, 2019)—a comprehensive package for structural equation modeling. An example of a basic EFA model using lavaan syntax with three latent variables and nine observed variables is the following:

efa(‘‘block1’’)*F1 =∼ x1 + x2 + x3 + x4 + x5 + x6 + x7 + x8 + x9
efa(‘‘block1’’)*F2 =∼ x1 + x2 + x3 + x4 + x5 + x6 + x7 + x8 + x9
efa(‘‘block1’’)*F3 =∼ x1 + x2 + x3 + x4 + x5 + x6 + x7 + x8 + x9

**Figure 2.**
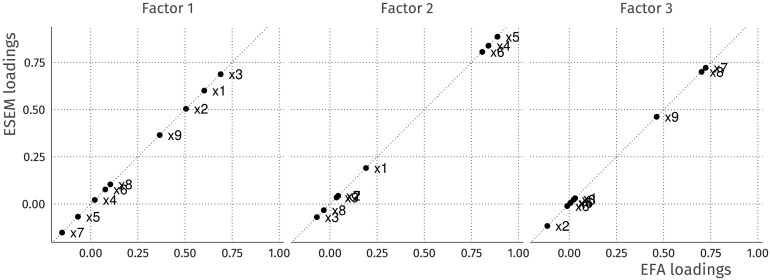
Exploratory factor analysis of nine variables in the Holzinger and Swineford (1939) dataset. On the *y*-axis are the estimated factor loadings using the oblimin rotation functionality in lavaan version 0.6-4, and the loadings on the *x*-axis are derived from factanal with oblimin rotation from the GPArotation package (Bernaards & Jennrich, 2005). The loadings are all on the diagonal with a correlation of 1, meaning the solutions obtained from these different methods are equal.

In effect, this model specifies three latent variables (F1, F2, and F3) which are each indicated by all nine observed variables (x1 to x9). The efa(‘‘block1’’) part is a modifier for this model which imposes the constraints on **Ψ** and **Λ** mentioned above. For a more detailed explanation of the lavaan syntax, see Rosseel (2012). Figure 2 shows a comparison of the factor loadings obtained using conventional factor analysis (factanal() in R) and lavaan’s efa() modifier. As shown, the solution obtained is exactly the same, with perfect correlation among the loadings for each of the factors.

With this tool as the basis for model estimation, the next section provides a detailed development of the construction of EFAST models.

### EFAST Models

We propose using EFA with corrections for contralateral covariance within the ESEM framework. The corrections we propose are the same as in MTMM models or CFA with residual covariance. In EFAST the method factors use CFA, and the remaining correlations are explained by EFA. Thus, unlike standard MTMM methods, EFAST contains *exploratory* factor analysis on the trait side, as the factor structure of the traits is unknown beforehand: the goal of the analysis is to extract an underlying low-dimensional set of features which explain the observed correlations as well as possible. For our running example of brain imaging data with contralateral symmetry, we consider each region of interest (ROI) a “method” factor, loading on only two regions. Note that in the context of brain imaging, Lövdén et al. (2013, Figure 1, model A) have had similar ideas, but their factor analysis operates on the level of left-right combined ROIs rather than individual ROIs.

**Figure 3.**
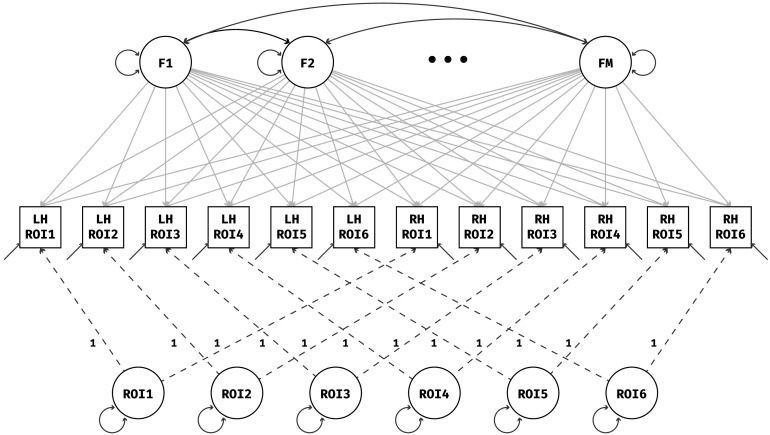
EFAST model with morphology of six regions of interest measured in the left hemisphere (LH) and right hemisphere (RH). The dashed lines indicate fixed loadings, and the two-headed arrows indicate variance/covariance parameters. The method factors are constrained to be orthogonal, and the loadings of the *M* traits are estimated in an exploratory way.

The EFAST model has *M* exploratory factors in a single EFA block, and one method factor per homologous ROI pair, each with loadings constrained to 1 and its own variance estimated. The estimated variance of the method factors then represents the amount of covariance due to symmetry, over and above the covariance represented by the traits. In Figure 3, the model is displayed graphically for a simplified example with six ROIs in each hemisphere.

An alternative parametrization for this model is also available. Specifically, we can use the correlations between the residuals of the observed variables instead of method factors with freely estimated variances. In the SEM framework, this would amount to moving the symmetry structure from the factor-explained matrix (**ΛΨΛ**^*T*^) to the residual covariance matrix **Θ**. This model is exactly equivalent, meaning the same correlation matrix decomposition, the same factor structure, and the same model fit will be obtained. However, we favour the method factor parametrization as it is closer to MTMM-style models, it is easier to extract potentially relevant metrics such as a “lateralisation coefficient,” and easier to extend to other data situations where multiple indicators load on each method factor.

To implement the EFAST model we use the package lavaan, which allows for easy scaling of the input data, different estimation methods, missing data handling through full information maximum likelihood, and more. Estimation of the model in Figure 3 can be done with a variety of methods. Here we use the default maximum likelihood estimation method as implemented in lavaan. Accompanying this paper, we are making available a convenient R package called efast that can fit EFAST models for datasets with residual structure due to symmetry. For more implementation details, the package and its documentation can be found at https://github.com/vankesteren/efast.

In the next section, we show how our implementation of EFAST compares to regular EFA in terms of factor loading estimation, factor covariance estimation, as well as the estimated number of factors.

## SIMULATIONS

In this section, we use simulated data to examine different properties of EFAST models when compared to regular EFA in controlled conditions. The purpose of this simulation is not an exhaustive investigation, but rather a pragmatically focused study of data properties (neuro) scientists wishing to use this technique are likely to encounter. First, we explain how data were simulated to follow a specific correlation structure, approximating the general structure of empirical data such as that in the Cam-CAN study (see Empirical Examples section). Then, we investigate the effects of structured residuals on the extracted factors from EFA and EFAST: in several different conditions, we investigate how the estimation of factor loadings, the covariances between factors, and the number of factors changes with increasing symmetry.

### Data Generation

**Figure 4.**
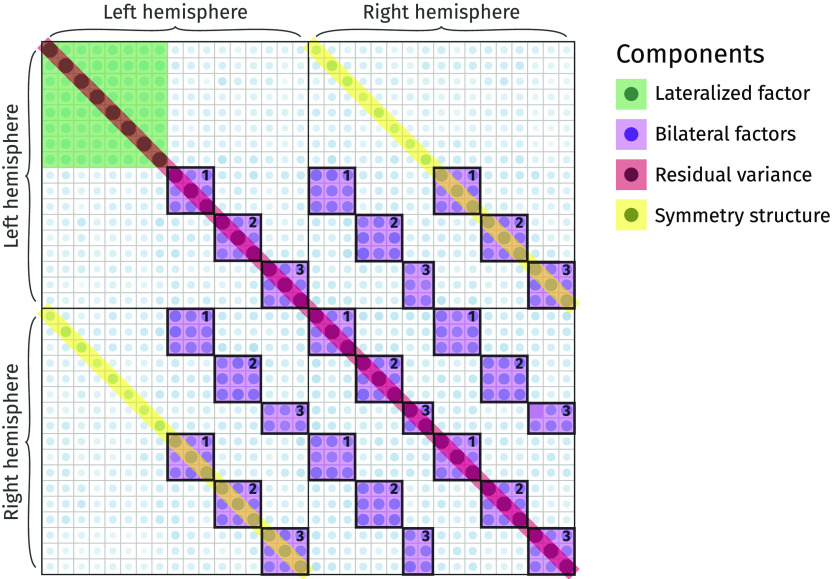
Example covariance matrix of the data-generating mechanism used in the simulations. This matrix results from simulated data of 650 brain images, with a factor loading of .595 for the lateralized factor, a loading of .7 for the remaining factors, a factor correlation of .5, and a symmetry correlation of .2. The first 17 variables indicate regions of interest (ROIs) in the left hemisphere, and the remaining variables indicate their contralateral homologues. Note the secondary diagonals, indicating contralateral symmetry, and the block of eight variables in the top left resulting from the lateralized factor.

Data were generated following a controlled population correlation matrix **Σ**_*true*_. This matrix represents the true correlation between measurements of brain structure in 17 left-hemisphere and 17 right-hemisphere ROIs. An example correlation matrix from our data-generating mechanism is shown in Figure 4.

**Figure 5.**
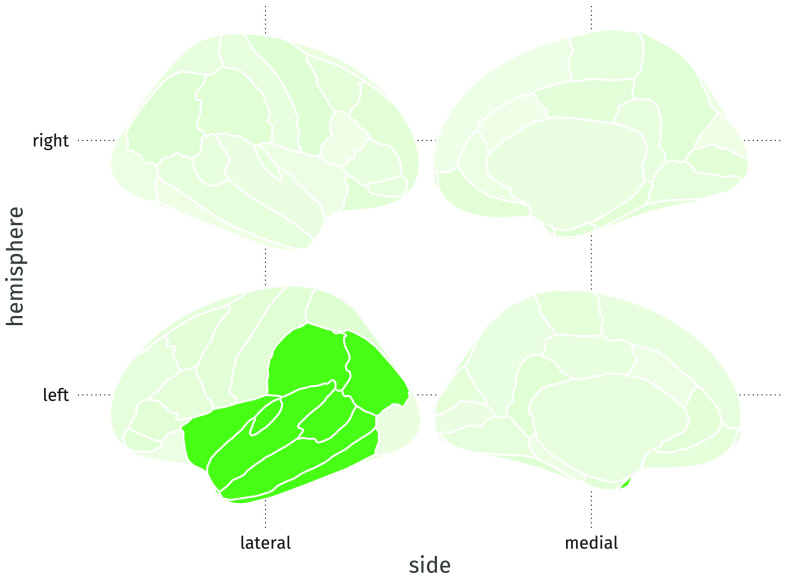
Example lateralized factor (the first factor in the simulation). Grey matter volume in eight left-hemisphere regions of interest are predicted by the value on this factor.

**Σ**_*true*_ was constructed through the summation of three separate matrices, as in the lower panel of Figure 1:

1.  The factor component **Σ**_*factor*_ is constructed as **ΛΨΛ**^*T*^, where the underlying factor covariance matrix **Ψ** can be either an identity matrix (orthogonal factors) or a matrix with nonzero off-diagonal elements (oblique factors). There are four true underlying factors in this simulation. One of the factors is completely lateralized (top left, highlighted in green), meaning that it loads only on ROIs in the left hemisphere. An additional illustration of this left-hemisphere factor is shown in Figure 5. The remaining three factors have both left- and right-hemisphere indicators.
2.  The structure component matrix is a matrix with all 0 elements except on the secondary diagonal, that is, the diagonal elements of the bottom left and top right quadrant are nonzero. The values of these secondary diagonals determine the strength of the symmetry.
3.  The residual variance component matrix is a diagonal matrix where the elements are chosen such that the diagonal of **Σ**_*true*_ is **1**.

For the following sections, data were generated with a sample size of 650, 130, or 65, a latent correlation of either 0 or 0.5, bilateral factor loadings of 0.5 or 0.7, lateral factor loadings of .425 or .595, and contralateral homology correlations of either 0 (pure EFA), 0.2 (minor symmetry), or 0.4 (major symmetry). These conditions were chosen to be plausible scenarios, similar to the observed data from our empirical examples. In each condition, 120 datasets were generated on which EFA and EFAST models with four factors were estimated. Thus, in each analysis the true number of factors is correctly specified before estimation. In the last simulation we then explore different criteria for the choice of number of factors in the case of contralateral symmetry.

### Effect of Structured Residuals on Factor Loadings

In this section, we compare estimated factor loadings from EFA and EFAST to the true factor loadings from the simulation’s data-generating process. For each condition, 120 datasets were generated, to which both EFA and EFAST models were fit. The factor loading matrix for each model was then extracted, the columns reordered to best fit the true matrix, and the mean absolute error of the factor loadings per factor was calculated.

**Figure 6.**
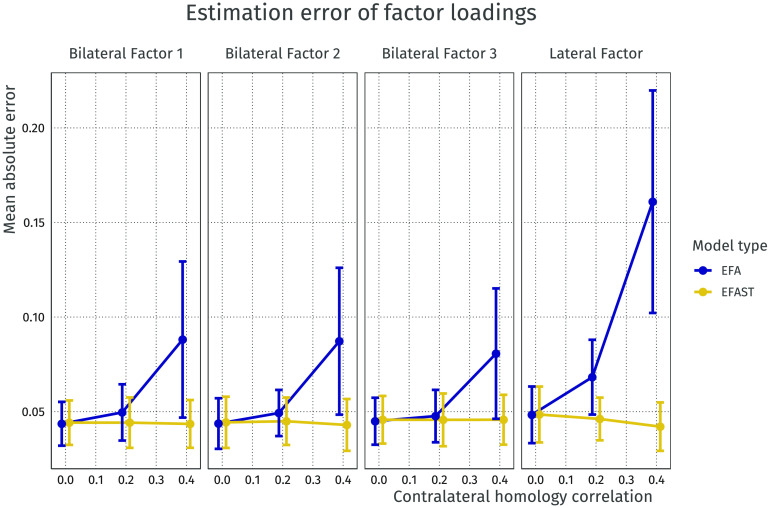
Mean absolute error for factor loadings of EFA versus EFAST models with increasing amounts of contralateral symmetry correlation. This plot comes from the condition where the sample size is 650, the covariance of the latent variables is 0.5, and the factor loadings are 0.5. The plot shows that for both bilateral and lateralised factors, EFA starts to exhibit more error as symmetry increases, more so for the lateral factor, whereas EFAST performance is nominal over these conditions. Error bars indicate 95% Wald-type confidence intervals.

As hypothesized, allowing structured residuals affects how well the factor loadings are estimated from the datasets. Notably, as shown in Figure 6 when performing regular EFA, the estimation error of the factor loadings increases when the symmetry becomes stronger, whereas the factor loading estimation error for the EFAST model remains at the level of regular EFA when there is no symmetry. Looking at the lateralized factor in particular, the adverse effect of omitting symmetry in dimension reduction becomes even stronger: in EFA, the lateralized factor becomes bilateral, leading to a larger error and an incorrect inference regarding the nature of the thus estimated factor. Although Figure 6 shows only the condition with a sample size of 650, factor loadings of 0.5, and factor covariance of 0.5, the pattern is similar for different sample sizes, different factor loading strengths, and with no factor covariance (see Supporting Information Figures S2 and S3).

In addition, sample size analysis shows that EFAST and EFA show moderate to high convergence rates for small (65) to moderate (130) sample sizes (see Supporting Information Figure S4). Although other drawbacks of smaller sample sizes remain (e.g., imprecise estimates, favouring of insufficiently complex models), this shows the feasibility, in principle, of using such analyses in commonly available sample sizes. To assess whether a particular combination of sample size, atlas dimensionality (i.e., number of regions) and strength of factor loadings is feasible for analysis using EFAST, we recommend a simulation approach. Software packages such as lavaan offer versatile tools to generate data under various specifications, allowing researchers to see whether a particular analysis is in principle feasible under certain idealized conditions before proceeding with real data.

Results from this section suggest that for the purpose of factor loading estimation, EFA and EFAST perform equally well in the case where a model without residual structure is the true underlying model, but EFAST outperforms EFA when residual structure in the observed data becomes stronger. In other words, implementing EFAST in the absence of residual structure does not seem to have negative consequences for estimation error, suggesting it may also be a useful default if a specific residual structure is thought, but not known, to exist. This is in line with Cole, Ciesla, and Steiger (2007), who argue that in many situations including correlated residuals does not have adverse effects, but omitting them does.

### Effect of Structured Residuals on Factor Covariances

Here, we compare how well EFA and EFAST retrieve the true factor covariance values. For both methods, we used geomin rotation with an epsilon value of 0.01 as implemented in lavaan 0.6.4 (Rosseel, 2019). The matrix product of the obtained rotation matrix ***H*** then represents the estimated factor covariance structure of the EFA factors: **Ψ**_*EFA*_ = ***H***^*T*^***H*** (Asparouhov & Muthén, 2009, eq. 22).

**Figure 7.**
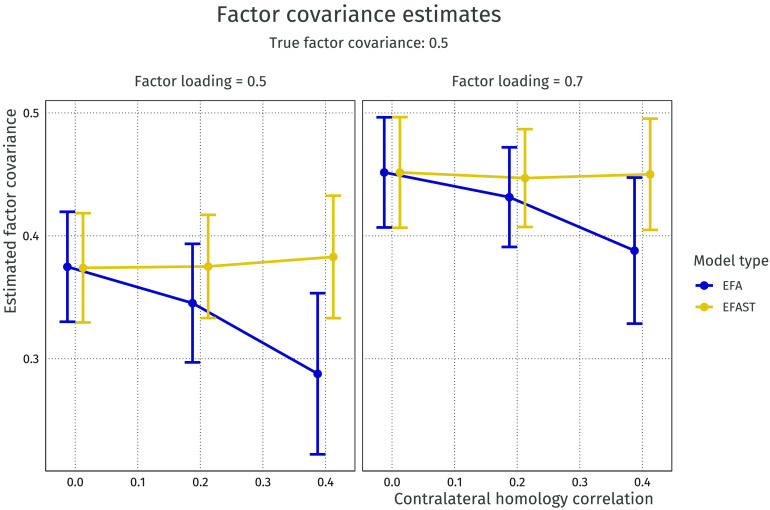
Latent covariance estimates for different levels of contralateral homology correlation. The true underlying latent covariance is 0.5; both methods underestimate the latent covariance but EFA becomes more biased as symmetry increases. Error bars indicate 95% Wald-type confidence intervals.

The mean of the off-diagonal elements of the **Ψ**_*EFA*_ matrix were then compared to the true value of 0.5 for increasing symmetry strength. The results are shown graphically in Figure 7. Here, it can be seen that with this rotation method the latent covariance is underestimated in all cases, although less so with stronger factor loadings. Furthermore, EFA performs worse as the symmetry increases, whereas the performance of EFAST remains stable regardless of the degree of contralateral homology, again suggesting no adverse effects to implementing EFAST in the absence of contralateral correlations. In the case of uncorrelated factors (not shown), the two methods perform similarly well.

The results from this section show that in addition to better factor recovery for EFAST, the recovery of factor covariance is also improved relative to EFA. Again, even when the data-generating mechanism does not contain symmetry, EFAST performs at least at the level of the EFA model. Note that in this case the overall model fit in terms of AIC and BIC is slightly better for the EFA model, as it has fewer parameters: for factor loadings of .5 and no symmetry, the mean AIC is 60148 (EFA) versus 60164 (EFAST), and BIC is 60882 (EFA) versus 60974 (EFAST). This, together with the comparable convergence rates for most conditions (Supporting Information Figure S4), suggests that it is viable to use EFAST as a “keep it maximal” strategy (Barr, Levy, Scheepers, & Tily, 2013), where EFAST can be used initially with no drawbacks, but one can use model evidence to favour classical EFA instead.

### Effect of Structured Residuals on Model Fit

In the above analyses, the number of factors was specified correctly for each model estimation (using either EFA or EFAST). However, in empirical applications the number of factors will rarely be known beforehand, so has to be decided on the basis of some criterion. A common approach to extracting the number of factors, aside from computationally expensive strategies such as parallel analysis (Horn, 1965), is model comparison through information criteria such as the AIC or BIC (e.g., Vrieze, 2012). In this procedure, models with increasing numbers of factors are estimated, and the best fitting model in terms of these criteria is chosen.

**Figure 8.**
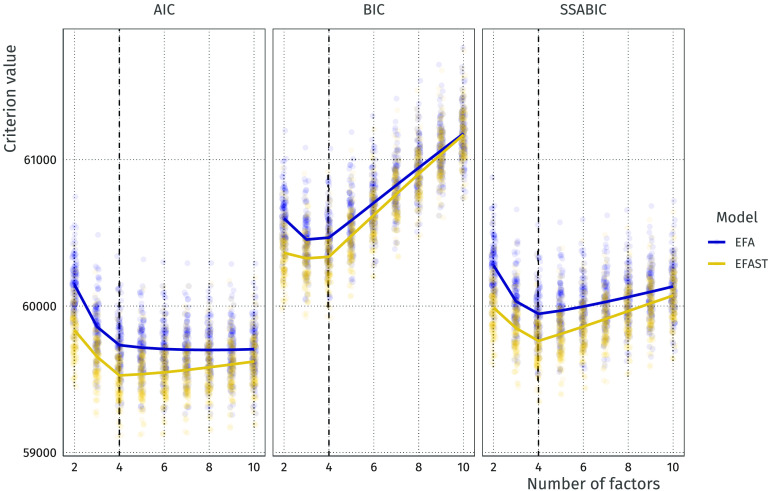
AIC and BIC values for increasing number of factors with EFA and EFAST models. Lines indicate expectations: the vertices are at the mean values for these criteria. The true number of factors is 4 (dashed vertical line).

In this simulation, we generated 100 datasets as in Figure 4, i.e., strong loadings and medium symmetry, and we fit EFA and EFAST models with 2 to 10 factors. Across these solutions we then compute the information criteria of interest. Here we choose the two most common information criteria (the AIC and BIC) as well as the sample size adjustedBIC (SSABIC), as this is the default in the ESEM function of the psych package (Revelle, 2018). The results of this procedure are shown in Figure 8. Each point indicates a fitted model. The means of the information criteria are indicated by the solid lines.

The plot in Figure 8 shows that across all factor solutions, EFAST shows better fit than EFA, suggesting the improvement in model fit outweighs the additionally estimated parameters. As the number of requested factors increases beyond optimality, this model fit improvement diminishes as EFA explains more of the symmetry structure through the additional factors. In general, the Akaike information criterion (AIC) tends to overextract factors, the Bayesian information criterion (BIC) slightly underextracts, and the SSABIC shows the best extraction performance (see also Supporting Information Figure S5). In practice, therefore, we suggest using SSABIC for determining the number of factors when model fit is of primary concern. Note that a researcher may also wish to determine the number of factors based on other considerations, such as usability in further analysis, estimation tractability, or theory.

## EFAST IN PRACTICE: MODELING BRAIN IMAGING DATA

In the field of cognitive neuroscience, a large body of work has demonstrated close ties between individual differences in brain structure and concurrent individual differences in cognitive performance such as intelligence tasks (e.g., Basten, Hilger, & Fiebach, 2015). Moreover, different aspects of brain structure can be sensitive to clinical and preclinical conditions such as grey matter for multiple sclerosis (Eshaghi et al., 2018), white matter hyperintensities for cardiovascular factors (Fuhrmann et al., 2019), and white matter microstructure for conditions such as ALS (Bede et al., 2015), Huntingtons (Rosas et al., 2010), and many other conditions.

However, one perennial challenge in imaging is how to deal with the dimensionality of imaging data. Depending on the spatial resolution, a brain image can be divided into as many as 100,000 individual regions, or voxels, rendering mass univariate approaches vulnerable to issues of multiple comparison. An alternative approach is to focus on sections called regions of interest (ROIs) defined either anatomically (e.g., Desikan et al., 2006) or functionally (e.g., Schaefer et al., 2018). However, this only solves the challenge of dimensionality in part, by grouping adjacent voxels into meaningful regions. An emerging approach is therefore to study how neural measures covary across populations or time, either in these ROIs (Sripada et al., 2019) or at the voxel level (DuPre & Spreng, 2017). This offers a promising strategy to reduce the high-dimensional differences in brain structure into a tractable number of components, or factors, not limited by spatial adjacency.

However, standard techniques such as EFA or PCA do not easily allow for the integration of a fundamental biological fact: that there exists strong contralateral symmetry between brain regions, such that any given region (e.g., the left lingual gyrus) is generally most similar to the same region on the other side of the brain. Here, we show how we can combine the strengths of exploratory data reduction with the integration of a priori knowledge about the brain into a more sensible, anatomically plausible factor structure which can either be pursued as an object of intrinsic interest or used as the basis for further investigations (e.g., which brain factors are most strongly associated with phenotypic outcomes).

### Empirical Example: Grey Matter Volume

#### Data Description

**Figure 9.**
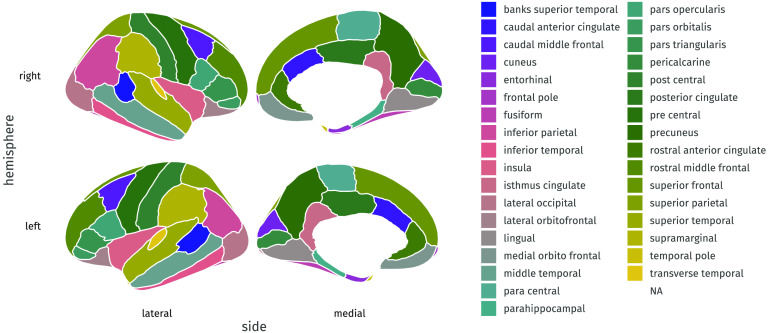
Desika–Killiany–Tourville atlas used in the empirical illustration, as included in the ggseg package (Mowinckel & Vidal-Piñeiro, 2019).

The data we use is drawn from the Cambridge Centre for Ageing and Neuroscience (Shafto et al., 2014; Taylor et al., 2017). Cam-CAN is a community derived lifespan sample (ages 18–88) of healthy individuals. Notably, the raw data from the Cam-CAN cohort is freely available through our data portal https://camcan-archive.mrc-cbu.cam.ac.uk/dataaccess/. The sample we discuss here is based on 647 individuals. For the purposes of this project we use morphometric brain measures derived from the T1 scans. Specifically, we used the Mindboggle pipeline (Klein et al., 2017) to estimate region-based grey matter volume, using the underlying freesurfer processing pipeline. To delineate the regions, we here use the Desikan–Killiany–Tourville atlas for determining the ROIs (Klein & Tourville, 2012) as illustrated in Figure 9.

**Figure 10.**
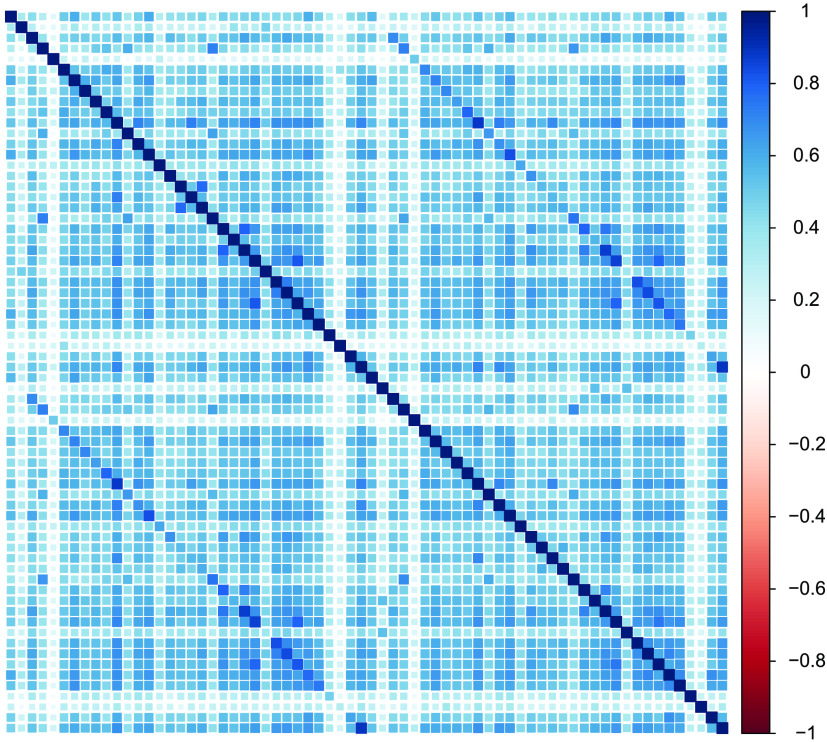
Correlation plot of cortical grey matter volume in 647 T1 weighted images of the Cam-CAN sample, estimated through Mindboggle in 34 brain regions in each hemisphere according to DKT segmentation. Numbers on the colour scale indicate the strength of the estimated correlation, with darker blue indicating stronger positive correlations. Secondary diagonal lines are visible indicating correlation due to contralateral homology.

We focus only on grey matter (not white matter) and only on cortical regions (not subcortical or miscellaneous regions such as ventricles) with the above atlas, for a total of 68 brain regions. The correlation matrix of regional volume metrics is shown in Figure 10, where the first 34 variables are ROIs in the left hemisphere, and the last 34 variables are ROIs in the right hemisphere. The presence of higher covariance due to contralateral homology is clearly visible in the darker secondary diagonal ‘stripes’ which show the higher covariance between the left/right version of each anatomical region. Our goal is to reduce this high-dimensional matrix into a tractable set of ‘brain factors,’ which we may then use in further analyses, such as differences in age sensitivity, in a way that respects known anatomical constraints.

**Figure 11.**
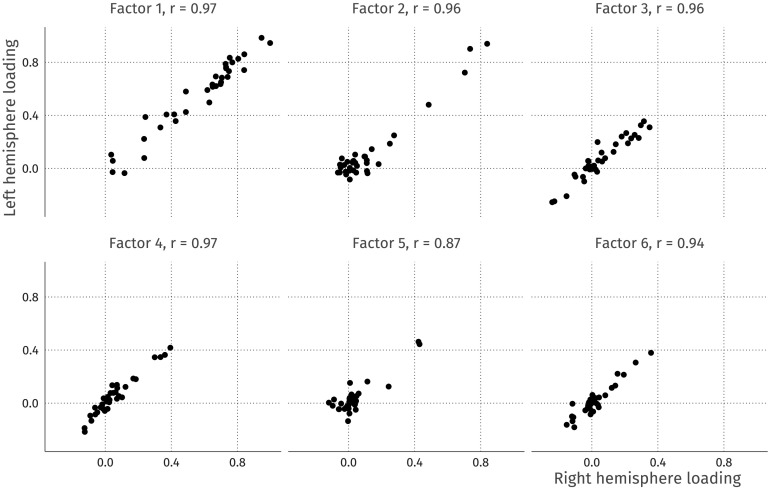
Left-right hemisphere factor loading correlations. The correlations between the loadings are high, indicating a strong similarity between the loadings in the left and right hemispheres.

The default estimation using EFA will attempt to account for the strong covariance among homologous regions seen in this data, meaning it is unlikely for, say, the left insula and the right insula to load on different factors, and/or for a factor to be characterized only/mostly by regions in one hemisphere. To illustrate this phenomenon, we first run a six-factor, geomin-rotated EFA for the above data (the BIC suggests six factors for this data using the EFAST model). The factor loadings for each ROI in the left and right hemispheres are plotted in Figure 11. A strong factor loading for a ROI in the left hemisphere is likely to have a strong factor loading in the right hemisphere due to the homologous correlation, as shown by the strong correlations for each of the factors.

In EFA, the resulting factors thus inevitably capture correlation due to contralateral symmetry, inflating or deflating factor loadings due to these contralateral residual correlations. Most problematically from a substantive neuroscientific standpoint, this distortion means it is effectively impossible to discover lateralized factors, that is, patterns of covariance among regions expressed only, or dominantly, in one hemisphere. This is undesirable, as there is both suggestive and conclusive evidence that some neuroscientific mechanisms may display asymmetry. For instance, typical language ability is associated with an asymmetry in focal brain regions (e.g., Bishop, 2013; Gauger, Lombardino, & Leonard, 1997), whereas structural differences in the right hemisphere may be more strongly associated with face perception mechanisms (Frässle et al., 2016). Developmentally, there is evidence that the degree of asymmetry changes across the lifespan (e.g., Plessen, Hugdahl, Bansal, Hao, & Peterson, 2014; Roe et al., 2020). Within a SEM context, recent work shows that model fit of a hypothesized covariance structure may differ substantially between the right and left hemispheres despite focusing on the same brain regions (Meyer, Garzón, Lövdén, & Hildebrandt, 2019). The ignorance of traditional techniques for the residual structure may cause lateralized covariance factors to appear symmetrical instead, or to not be observed at all.

#### Results

In this section, we compare the model fit and factor solutions of EFA and EFAST for the Cam-CAN data, and we show how EFAST decomposes the correlation matrix in Figure 10 into factor, structure, and residual variance components. The full annotated analysis script to reproduce these results is available as Supporting Information to this manuscript.

**Figure 12.**
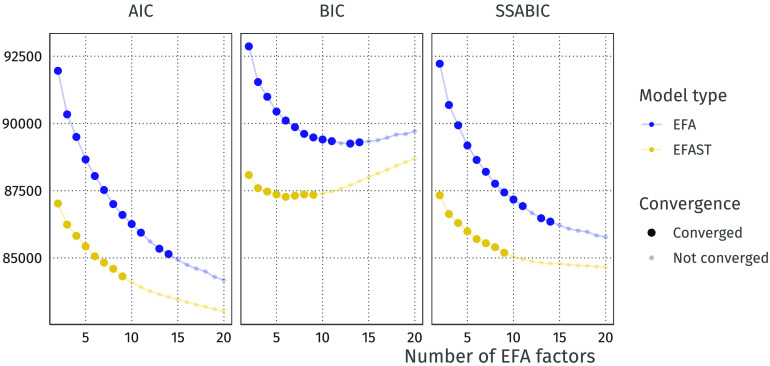
AIC and BIC for the increasing numbers of EFA factors. Semitransparent points indicate models which are inadmissible either due to nonconvergence or convergence to a solution with problems (e.g., Heywood cases). In these cases we plot the information criteria based on the log-likelihood computed at the time the estimation terminated.

Overall, the EFAST model performs considerably better than standard EFA using common information criteria (Figure 12). The BIC criterion, combined with the convergence of the models to an admissible solution, suggests that six factors is optimal for this dataset. While both AIC and SSABIC show that more factors may be needed to properly represent the data, we see that this quickly leads to nonconvergence. We here consider six factors to be a tractable number for further analysis. First and foremost, this six-factor solution shows a much better model solution under EFAST (BIC ≈ 87,500) than under EFA (BIC ≈ 90,000), emphasizing the empirical benefits of appropriately modeling known biological constraints. Additionally, statistical model comparison through a likelihood ratio test shows that the EFAST model fits significantly better (see Table 1). Other fit measures such as CFI, root-mean-square error of approximation (RMSEA), and standardized root mean residual (SRMR) paint a similar story. The full factor loading matrix for both EFAST and EFA are shown in Supporting Information Table S1.

**Table 1.** Comparing the fit of the EFAST and EFA models with six factors, using a likelihood ratio test and several fit criteria

	CFI	RMSEA	SRMR	*χ*^2^	Df	Δ*χ*^2^	ΔDf	Pr(> *χ*^2^)
EFAST	0.912	0.057	0.209	5762.676	1851			
EFA	0.843	0.075	0.342	8818.146	1885	3055.471	34	< .001

**Figure 13.**
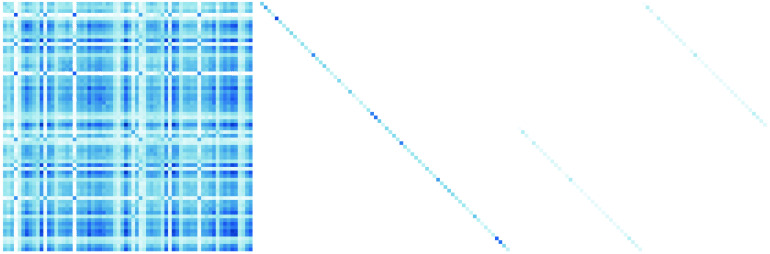
Extracted correlation matrix components using a six-factor EFAST model with unconstrained correlations. Darker blue indicates stronger positive correlation. From left to right: factor-implied correlations, residual variance, and structure matrix.

The EFAST model decomposes the observed correlation matrix from Figure 10 into the three components displayed in Figure 13. The most notable observation here is the separation of symmetry structure (last panel) and latent factor-implied structure (first panel): the factor solution (first panel) does not attempt to explain the symmetry structure seen in the data (i.e., the characteristic diagonal streaks are no longer present). This indicates that the EFAST model correctly separates symmetry covariance from underlying trait covariance in real-world data.

**Figure 14.**
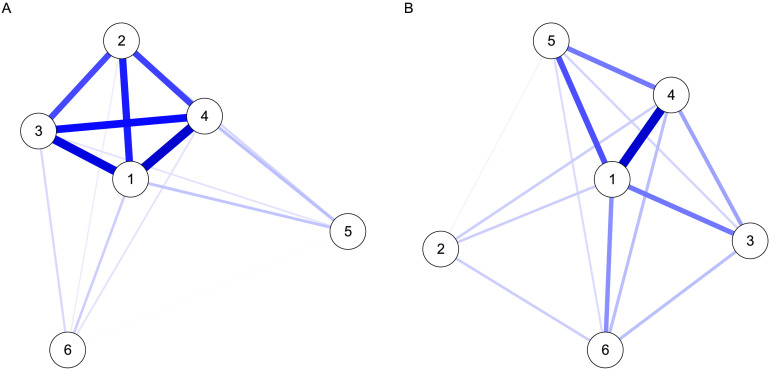
Network plots of the latent covariance for EFA (A) and EFAST (B).

We also extracted the estimated factor covariance, shown as a network plot in Figure 14. For EFA, some latent variables show very strong covariance, clustering them together due to the contralateral symmetry. This effect is not visible in the EFAST model, which shows a more well-separated latent covariance structure. This suggests that one consequence of a poorly specified EFA can be the considerable overestimation of factor covariance, which in turn adversely affects the opportunities to understand distinct causes or consequences of individual differences in these factors.

### Empirical Example: White Matter Microstructure

#### Data Description

**Figure 15.**
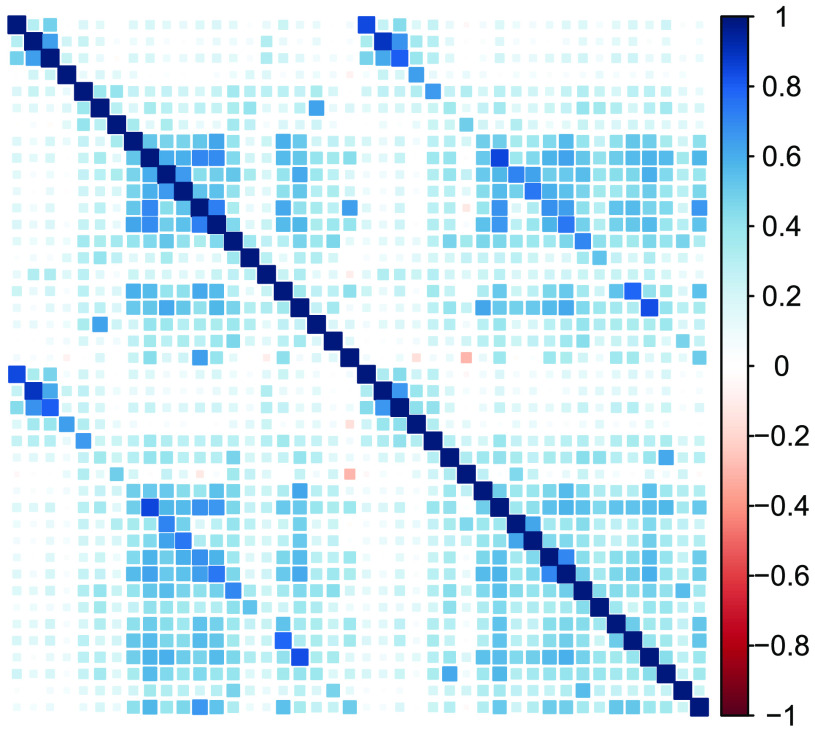
Correlation matrix for Cam-CAN white matter tractography data (fractional anisotropy). Numbers on the colour scale indicate the strength of the estimated correlation, with darker blue indicating stronger positive correlations. Secondary diagonal lines are visible indicating correlation due to contralateral homology.

Our second empirical example uses white matter structural covariance networks. We use 42 tracts from the ICBM-DTI-81 atlas (Mori et al., 2008), including only those tracts with atlas-separated left/right tracts (i.e., excluding divisions of the corpus callosum). As anatomical metric we use tract-based mean fractional anisotropy, a summary metric sensitive (but not specific) to several microstructural properties (Jones, Knösche, & Turner, 2013). The resulting correlation matrix is shown in Figure 15. For more details regarding the analysis pipeline, see Kievit et al. (2016). The same tracts and data were previously analysed in Jacobucci, Brandmaier, and Kievit (2019).

#### Results

We chose 6 factors for the EFAST and EFA models based on the SSABIC in combination with the convergence limitations. In Table 2, the two models are compared on various characteristics. From the likelihood ratio test, we can see that the EFAST model represents the white matter data significantly better (*χ*^2^(21) = 3632.586, *p* < .001), and inspecting the SSABIC values (EFA = 59,120, EFAST = 55,727) leads to the same conclusion. In addition, the CFI, RMSEA, indicate better fit for the EFAST model, too.

**Table 2.** Comparing the fit of the EFAST and EFA models with six factors for the white matter data, using a likelihood ratio test and several fit criteria

	CFI	RMSEA	SRMR	Df	*χ*^2^	Δ*χ*^2^	ΔDf	Pr(> *χ*^2^)
EFAST	0.899	0.081	0.205	603	3137.462			
EFA	0.756	0.123	0.198	624	6770.048	3632.586	21	< .001

**Figure 16.**
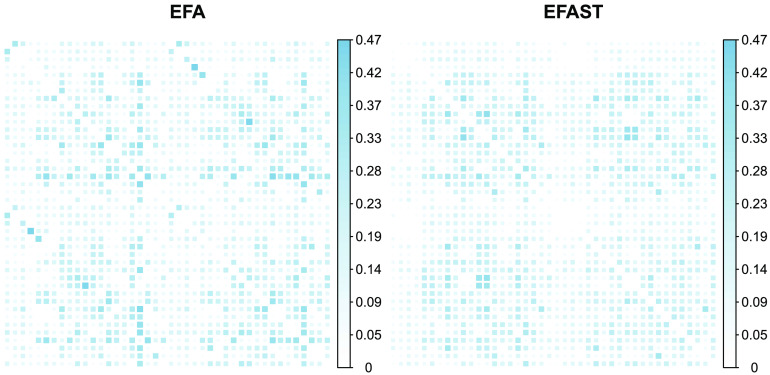
Visual representation of the root square residual (observed, implied) correlations, which form the basis of the SRMR fit index. Numbers on the colour scale indicate root square residual correlation, darker blue indicates larger residual.

The only index which indicates slightly poorer fit is the SRMR. The difference is very small in this case, but nonetheless it is relevant to show where these differences lie. A visual representation of the root square residual (observed, implied) correlations, which form the basis of the SRMR fit index, can be found in Figure 16. The figure shows that EFAST is able to represent the symmetry better: it has almost no residuals on the secondary diagonals. The remaining residuals are very similar, though slightly higher, leading to a higher SRMR.

### Empirical Example: Resting-State Functional Connectivity

#### Data Description

**Figure 17.**
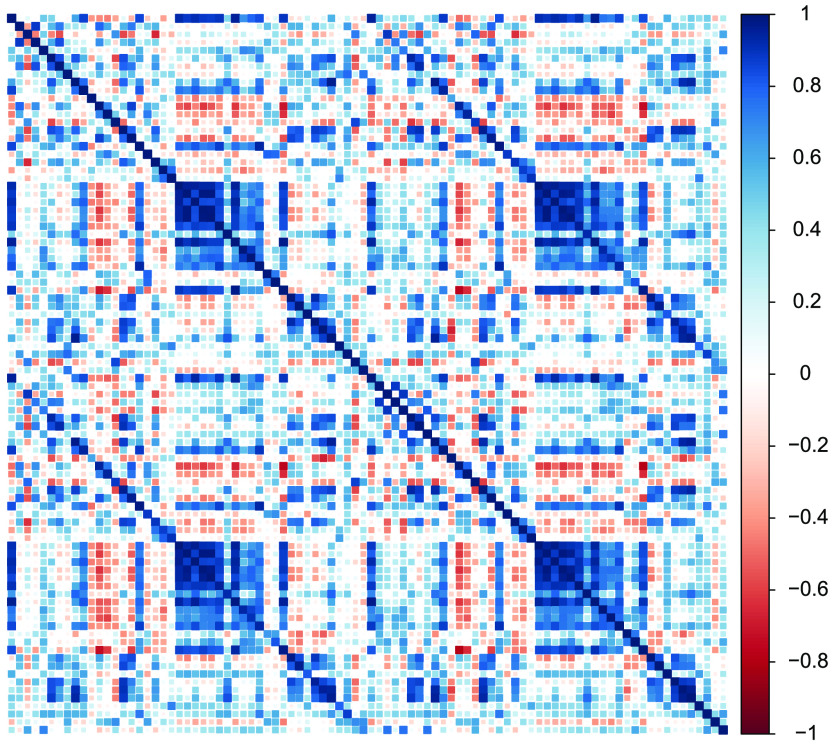
Correlation matrix for the first participant in the Cam-CAN resting-state functional connectivity dataset. Numbers on the colour scale indicate the strength of the estimated correlation, with darker blue indicating stronger positive correlations. Secondary diagonal lines are visible indicating correlation due to contralateral homology.

Our previous examples deal with correlation matrices capturing between-individual similarities across regions. However, the same techniques can be implemented at the within-subject level given suitable data. One such measure is *functional connectivity* which reflects the temporal connectivity between regions during rest or a given task, and captures the purported strength of interactions, or communications, between regions (Van Den Heuvel & Pol, 2010). Here we use functional connectivity matrices from five participants in the Cam-CAN study measured during an eyes-closed resting-state block. We focus on 90 cortical and subcortical regions from the AAL atlas (Tzourio-Mazoyer et al., 2002). The methodology to compute the connectivity metrics is outlined in (Geerligs, Tsvetanov, & Henson, 2017), and the data reported here have been used in Lehmann et al. (2019). The correlation matrix for the first participant is shown in Figure 17.

#### Results

For this example, data from the first participant was used to perform the model fit assessments. We performed a similar routine as with the previous empirical datasets for determining the number of factors: we fit the EFAST and EFA models for 2–16 factors and compare their information criteria. All of the models converged, and the optimal model based on the BIC is a 13-factor EFAST model. BIC was chosen as a criterion for the number of factors in order to keep the analysis tractable; the other criteria indicated an optimum beyond 16 factors.

The 13-factor EFAST model was then compared to the 13-factor EFA model on various fit indices. The results of this comparison can be found in Table 3. Across the board, the EFAST model has better fit, as the EFAST CFI, RMSEA, SRMR, and *χ*^2^ fit indices outperform those for the EFA model, demonstrating that accounting for the bilateral symmetry in dimension reduction through factor analysis leads to better fitting model of the data.

**Table 3.** Comparing the fit of the EFAST and EFA models with 13 factors for the functional resting-state data, using a likelihood ratio test and several fit criteria

	CFI	RMSEA	SRMR	Df	*χ*^2^	Δ*χ*^2^	ΔDf	Pr(> *χ*^2^)
EFAST	0.836	0.093	0.253	2868	9350.278			
EFA	0.774	0.108	0.272	2913	11828.126	2477.848	45	0.000

**Figure 18.**
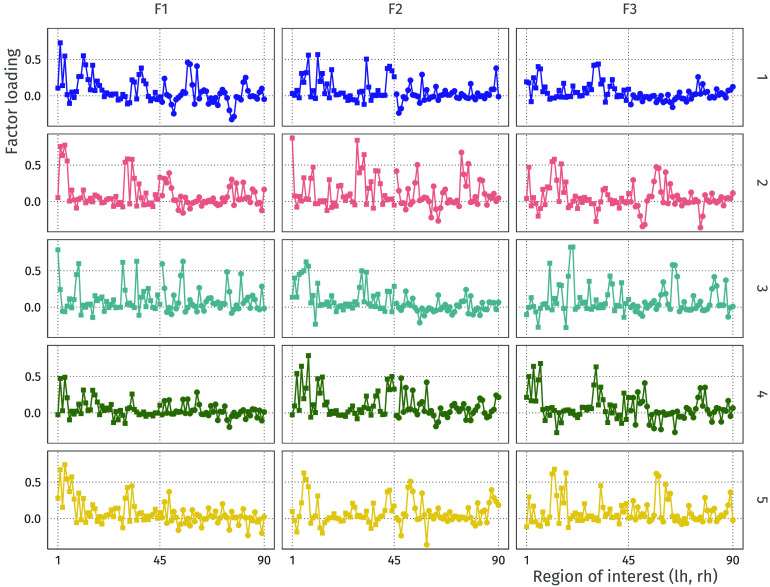
Comparison of factor loading profiles for the first three factors (columns) across five participants (rows). The left side of each subplot corresponds to the left hemisphere, and the right side corresponds to the right hemisphere.

This approach also allows for comparing the factor loadings for the different participants. For illustration, the plot in Figure 18 shows the profile of factor loadings for the first three factors (columns) across the five participants (rows). These profile plots can be a starting point for comparison of the connectivity structure across participants, where higher correlation among participants means a more similar connectivity structure, while taking into account the symmetry in the brain. For example, for Factor 1, participant 3 has a quite different functional connectivity factor loading profile than the other participants.

## MODEL-BASED LATERALIZATION INDEX

In the simulations, we showed how the EFAST approach yields a more veridical representation of the factor structure than EFA. However, using EFAST yields an additional benefit: our model allows for estimating the extent of symmetry in each ROI, while taking into account the overall factor structure. This enables researchers to use this component of the analysis for further study. The (lack of) symmetry may be of intrinsic interest, such as in language development research (Schuler et al., 2018), intelligence in elderly (Moodie et al., 2019), and age-related changes in cortical thickness asymmetry (Plessen et al., 2014). In the EFAST package, we have implemented a specific form of lateralization which is based on a variance decomposition in the ROIs. Our lateralization index (LI) is a dissimilarity measure representing the proportion of residual variance (given the trait factors) in an ROI that cannot be explained by symmetry. The index value is 0 if the bilateral ROIs are fully symmetric (conditional on the trait factors), and 1 if there is no symmetry:

$$LI_{i}=1-\text{cor}(u_{i}^{lh},u_{i}^{rh})$$ (1)

where $u_{i}^{lh}$ and $u_{i}^{rh}$ are residuals given the trait factors of interest of the *i*^*th*^ ROI in the left and right hemisphere, respectively. The correlation cor(⋅, ⋅) between these residuals represents the amount of symmetry, so the *LI*_*i*_ represents the *residual dissimilarity* of the *i*^*th*^ ROI in the two hemispheres after taking into account the factor structure in the data. When *LI*_*i*_ is 0, the ROIs are fully symmetric given the traits, and a *LI*_*i*_ of 1 indicates no symmetry. Note that *LI*_*i*_ can be larger than 1 if the residuals are negatively correlated.

**Figure 19.**
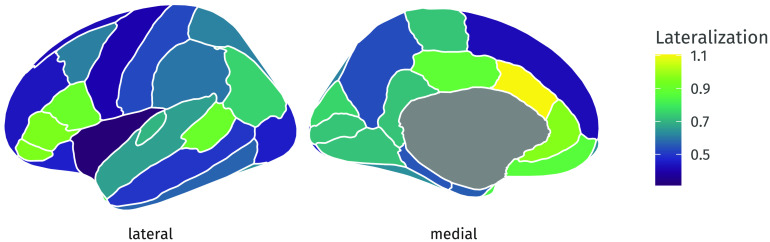
Amount of grey matter volume asymmetry per ROI. Dark blue areas are highly symmetric given the previously estimated 6-factor solution, and bright yellow areas are highly asymmetric. Such plots can be made and compared for different groups and statistically investigated for differences in symmetry for a common factor solution. A lateralization index (LI) of 0 means that the regions are fully symmetric conditional on the trait factors.

**Figure 20.**
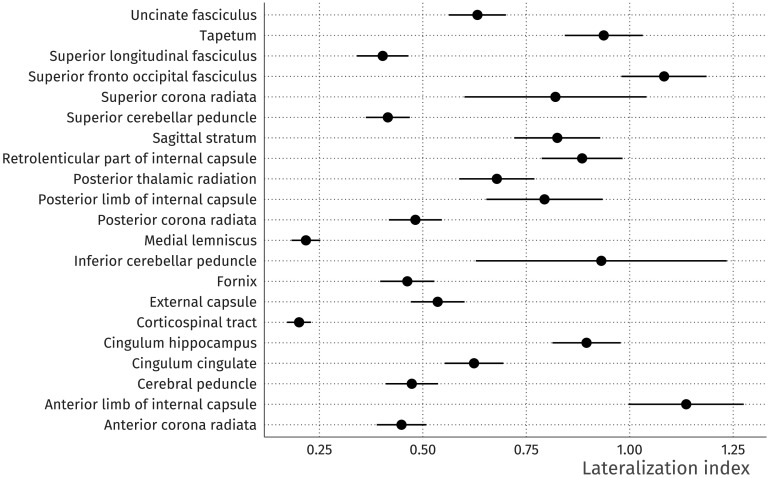
White matter lateralization index for a selected set of regions given the previously estimated 6-factor solution. Lower values mean that bilateral ROIs are more symmetric conditional on the trait factors, higher values that they are less so. The line ranges indicate 95% confidence intervals, computed as *LI* ± 1.96 × *SE*_*LI*_, where the standard error *SE*_*LI*_ is computed using the delta method.

The LI for each ROI in the grey matter volume example is shown in Figure 19. Here, we can see that there is high lateralization in the superior temporal sulcus and medial orbitofrontal cortex, but high symmetry in the lateral orbitofrontal cortex and the insula. In Figure 20, we additionally show in the white matter example that LI can naturally be supplemented by standard errors and confidence intervals. Thus, the EFAST procedure not only improves the factor solution under plausible circumstances for such datasets, but in doing so yields an intrinsically interesting metric of symmetry.

## SUMMARY AND DISCUSSION

In this paper, we have developed and implemented EFAST, a method for performing dimension reduction with residual structure. We show how this new method outperforms standard EFA across three separate datasets, by taking into account hemispheric symmetry in brain covariance data. We have argued through both simulations and real-world data analysis that our method is an improvement in the dimension reduction step of such high-dimensional, structured data, yielding a more veridical factor solution. Such a factor solution can be the basis for further analysis, such as an extension of the factor model to prediction of continuous phenotype variables such as intelligence scores, or the comparison among different age groups. These extensions will be improved by building on a factor solution which appropriately takes into account the symmetry of the brain. Furthermore, we believe that many data reduction problems in social, cognitive, and behavioural sciences have a similar structure: residual structure is known, but precise theory about the underlying factor structure is not (Asparouhov & Muthén, 2009). As such, although we focus on brain imaging data, our approach is likely more widely applicable.

Care is needed in the interpretation of the factor solution as underlying dimensions, as the empirical application has shown that the absolute level of fit for both the EFA and EFAST models is not optimal. In addition, estimation of more complex factor models may lead to nonconvergence or inadmissible solutions. Such problems would need to be further investigated, potentially leading to more stable estimation, for example, through a form of principal axis factoring, or potentially through penalization of SEM (Jacobucci et al., 2019; van Kesteren & Oberski, 2019). However, these limitations hold equally for EFA, and when comparing both methods it is clear from the results in this paper that the inclusion of structured residuals greatly improves the representation of the high-dimensional raw data by the low-dimensional factors. In summary, this relatively simple but versatile extension of classical EFA may be of considerable value to applied researchers with data that possess similar qualities to those outlined above. We hope our tool will allow those researchers to easily and flexibly specify and fit such models.

Note that we are not the first to suggest using structured residuals in EFA to take into account prior knowledge about structure in the observed variables. Adding covariances among residuals is a common method to take into account features of the data-generating process (e.g., Cole et al., 2007), and this has been possible in the context of EFA since the release of the ESEM capability in MPlus (Asparouhov & Muthén, 2009) and in lavaan (Rosseel, 2019). In the context of neuroscientific data, similar methods in accounting for structure in dimension reduction have been researched by De Munck, Huizenga, Waldorp, and Heethaar (2002) in source localization for EEG/MEG. Our goal for this paper has been to provide a compelling argument for the use of such structured residuals from the point of view of neuroscience, as well as a user-friendly, open-source implementation of this method for dimension reduction in real-world datasets.

## ACKNOWLEDGMENTS

We would like to thank Yves Rosseel for valuable input and the development of key tools, Linda Geerligs for providing the functional connectivity data, and Jonathan Helm and Øystein Sørensen for their helpful comments on an earlier version of this manuscript.

## SUPPORTING INFORMATION

Supporting information for this article is available at https://www.doi.org/10.1162/00157, https://doi.org/10.5281/zenodo.3779927 (van Kesteren & Kievit, 2020a), and https://doi.org/10.5281/zenodo.3779908 (van Kesteren & Kievit, 2020b).

## AUTHOR CONTRIBUTIONS

Erik-Jan van Kesteren: Conceptualization; Formal analysis; Investigation; Methodology; Software; Visualization; Writing - Original Draft; Writing - Review & Editing. Rogier A. Kievit: Conceptualization; Data curation; Investigation; Methodology; Project administration; Resources; Supervision; Writing - Review & Editing.

## FUNDING INFORMATION

Erik-Jan van Kesteren, Nederlandse Organisatie voor Wetenschappelijk Onderzoek (http://dx.doi.org/10.13039/501100003246), Award ID: 406.17.057. Rogier A. Kievit, Medical Research Council (http://dx.doi.org/10.13039/501100000265), Award ID: SUAG/047 G101400. Rogier A. Kievit, Horizon 2020 (http://dx.doi.org/10.13039/501100007601), Award ID: 732592.

## TECHNICAL TERMS

Dimension reduction: Summarizing a set of variables by using a smaller set of components or factors.
Structural equation model: Method for estimating latent variables and their relations by using observed covariances, assuming linearity and normally distributed residuals.
Confirmatory factor analysis: Factor model where the factor loading structure is predefined based on theory; typically, each factor only predicts a subset of the observed variables.
Multitrait-multimethod model: Factor model which also assumes there is residual covariance between some variables because they are measured using the same method.
Residual covariance: Covariance among unexplained parts of variables, after taking into account the model (in this case the factors).
Factor loadings: Weights matrix indicating the strength of the association between each latent factor and each observed variable.
Mean absolute error: In a simulation study, the absolute difference between the estimated and the true parameter values.
Information criterion: An indicator weighing model fit (likelihood) and model complexity (number of parameters); lower values indicate better models.
RMSEA: Root-mean-square error of approximation, a popular model fit index based on the log-likelihood and the degrees of freedom of a structural equation model. Values closer to 0 indicate better fit.
SRMR: Standardized root mean residual, a value indicating how well a predicted correlation matrix approximates the observed correlation matrix. Values closer to 0 indicate better fit.
