# Supplementary material for: Exploratory factor analysis with structured residuals for brain network data
Source: Netw Neurosci. 2021 Feb 1;5(1):1–27. doi: 10.1162/netn_a_00162 (PMC7935039; doi:10.1162/netn_a_00162)
Supplement: Supplementary file 1 [file netn-05-1-s001.pdf]

van Kesteren, E. & Kievit, R. A. (2021). Supporting information for "Exploratory Factor Analysis with Structured Residuals for Brain Network Data." *Network Neuroscience*, 5(1), 1–27. https://doi.org/10.1162/netn_a_00162

Supplementary Figures and Tables

Exploratory Factor Analysis with Structured Residuals for Brain Network Data

Erik-Jan van Kesteren Rogier A. Kievit

July 14, 2020

1 Symmetry pattern recovery with default EFA

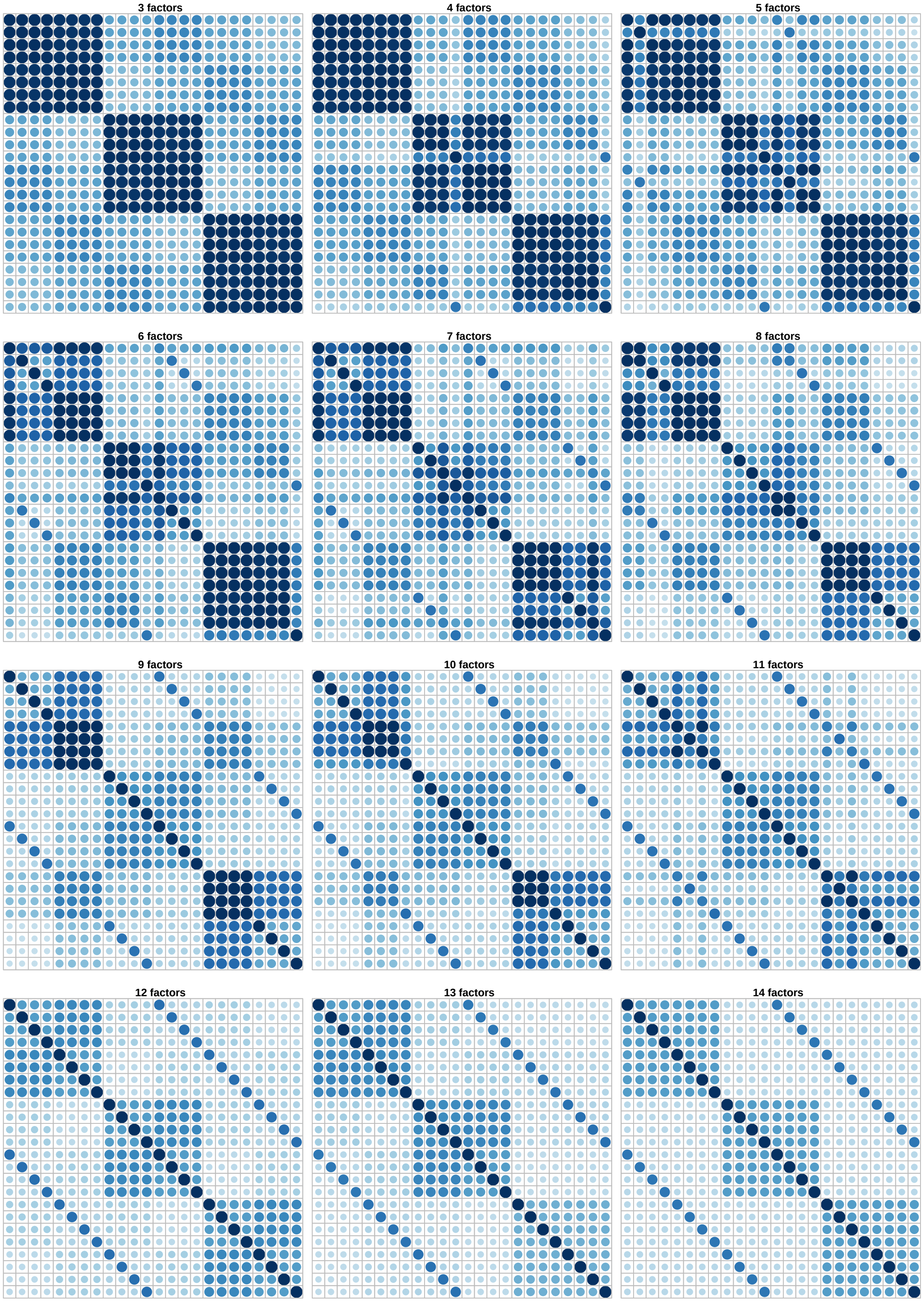

Figure S1: Predicted correlation matrix for EFA models with M factors for the example observed correlation matrix of Figure 1 in the main text. Proper recovery of the observed pattern happens around 12 factors (bottom left frame).

2 Comparing EFA and EFAST in factor loading estimation error

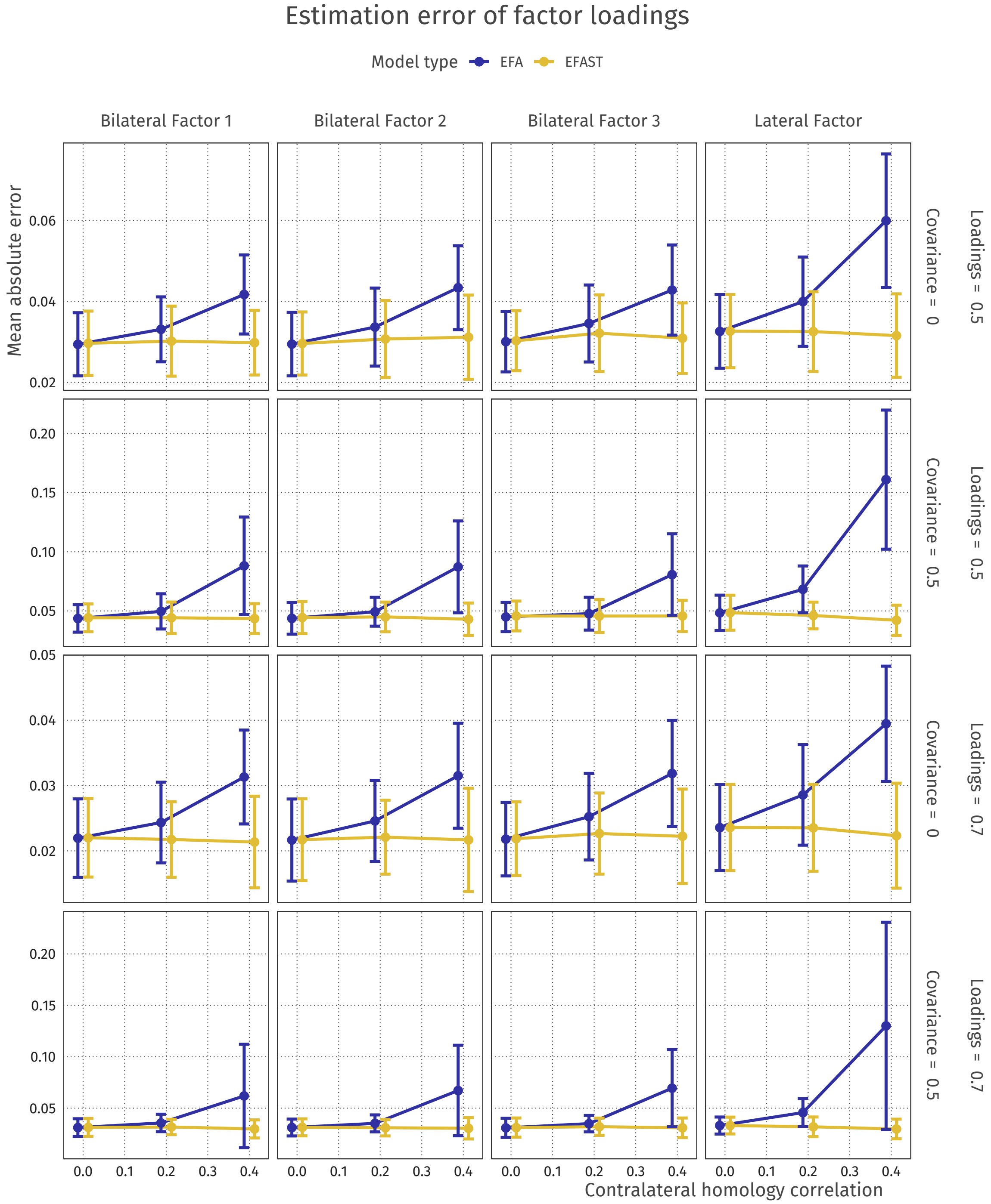

Figure S2: Factor loading median absolute error over different conditions of factor loading and factor correlation strength (top-to-bottom, see labels on the right) and different factors (left-to-right, see labels on top).

3 Sample size in factor loading estimation error

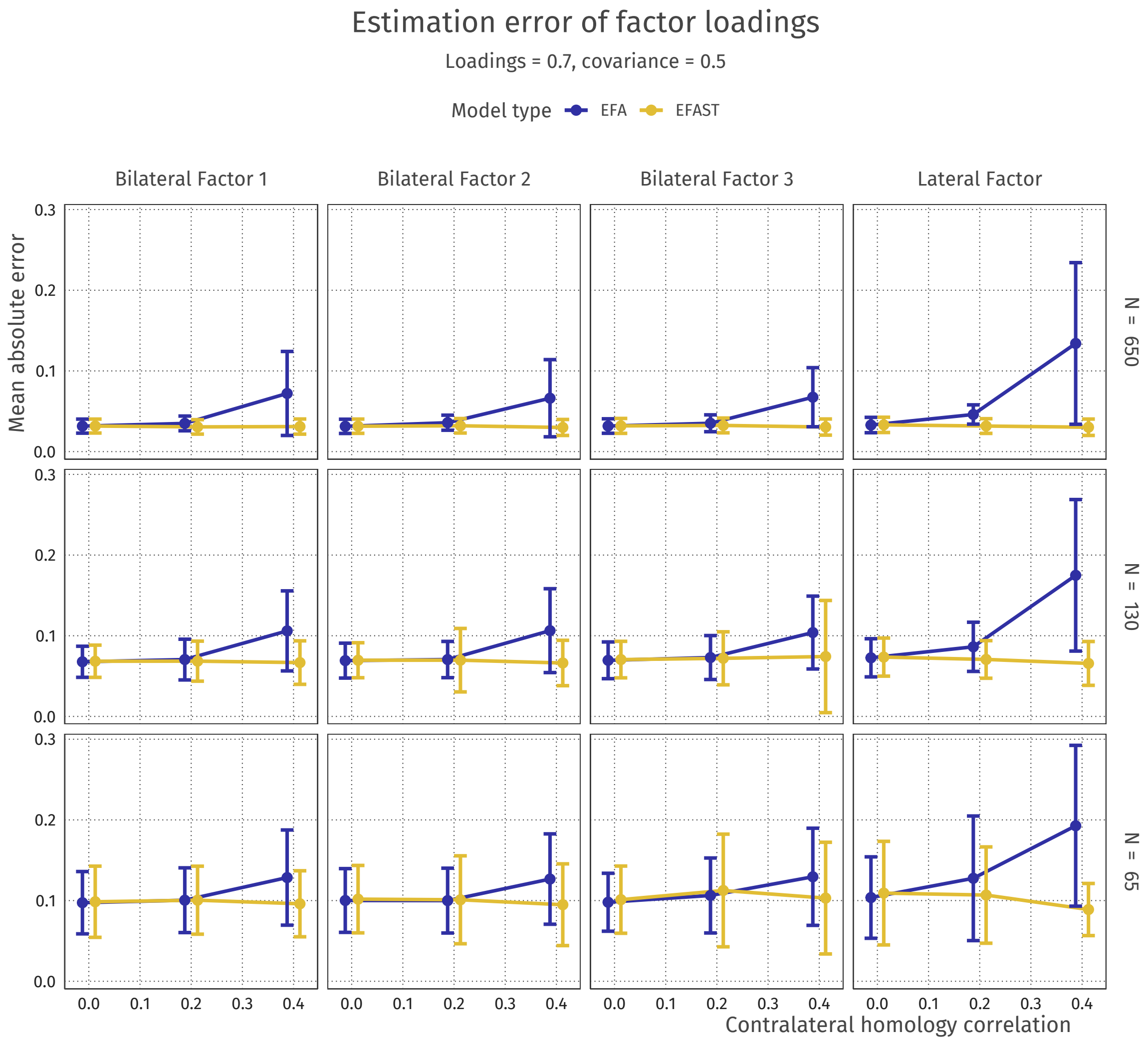

Figure S3: Factor loading median absolute error over different sample sizes (top-to-bottom, see labels on the right) and different factors (left-to-right, see labels on top).

4 Sample size and model estimation convergence

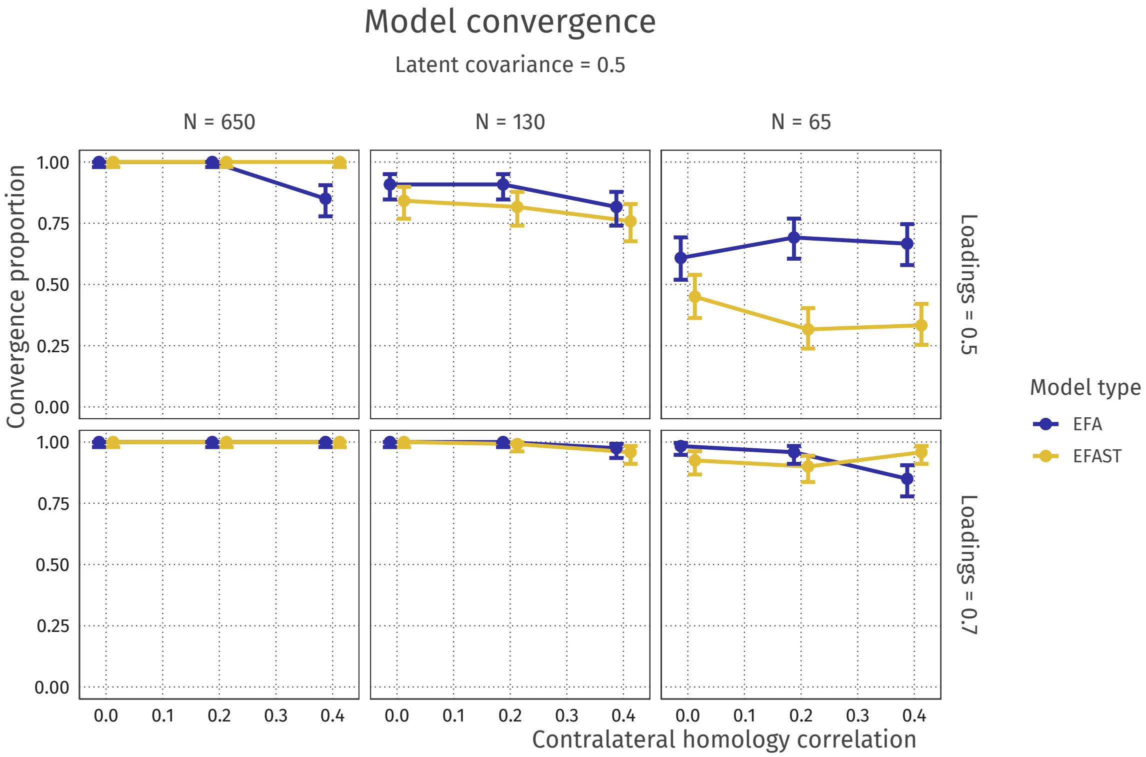

Figure S4: Convergence rates of EFA and EFAST for different sample sizes (left-to-right, see labels on top). Convergence probability is not only determined by the sample size, but also by other factors such as the amount of latent covariance, the strength of the factor loadings, and the amount of symmetry.

5 Information criterion factor extraction performance

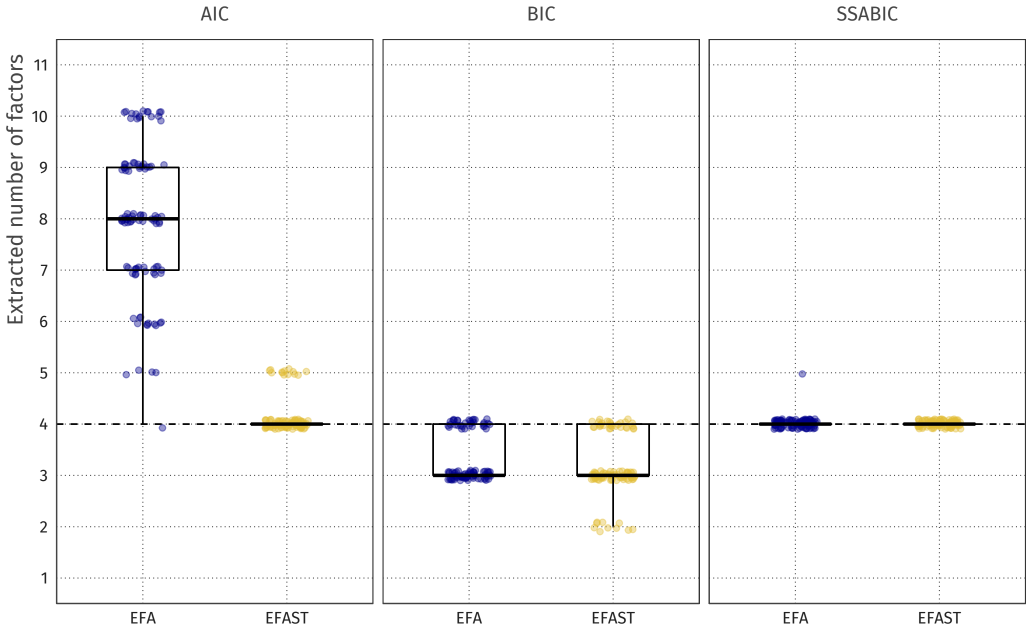

Figure S5: Number of extracted factors using the AIC (left panel), BIC (middle panel), and sample-size adjusted BIC (right panel) criterion. AIC works well for the EFAST method but not for the EFA method. BIC slightly underextracts for both methods. SSABIC shows excellent performance for both methods. The true number of factors is 4 (dashed line), for which this result holds; different simulation situations may show different factor extraction patterns.

6 Factor loadings for empirical application

Table S1: Factor loadings for 6-factor model fitted using EFAST and EFA on the Cam-CAN volume data. Loadings with absolute value below 0.3 not shown.

	EFAST						EFA					
	F1	F2	F3	F4	F5	F6	F1	F2	F3	F4	F5	F6
lh__bankssts	0.38				0.6		0.79					
lh__caudalanteriorcingulate				-0.4	0.55							
lh__caudalmiddlefrontal					0.73						0.74	
lh__cuneus		0.91						0.87				
lh__entorhinal			0.31									
lh__fusiform					0.4		0.38					
lh__inferiorparietal					0.69		0.67					
lh__inferiortemporal					0.4		0.54					
lh__isthmuscingulate					0.44							
lh__lateraloccipital		0.45					0.37	0.46				
lh__lateralorbitofrontal					0.78						0.71	
lh__lingual		0.71						0.7				
lh__medialorbitofrontal					0.65						0.63	
lh__middletemporal	0.31				0.66		0.81					
lh__parahippocampal					0.37							
lh__paracentral					0.83						0.73	
lh__parsopercularis					0.79						0.72	
lh__parsorbitalis					0.61						0.63	
lh__parstriangularis					0.84						0.89	
lh__pericalcarine		0.91						0.91				
lh__postcentral					0.8		0.37				0.33	
lh__posteriorcingulate					0.72						0.52	
lh__precentral			-0.3		0.87				-0.34		0.72	
lh__precuneus					0.76							0.74
lh__rostralanteriorcingulate					0.59						0.49	
lh__rostralmiddlefrontal					0.78						0.82	
lh__superiorfrontal					0.88						0.93	
lh__superiorparietal					0.72							0.77
lh__superiortemporal					0.77		0.52				0.41	
lh__supramarginal					0.77		0.32					
lh__frontalpole					0.32						0.51	
lh__temporalpole			0.39						0.34			
lh__transversetemporal					0.72					0.43	0.53	
lh__insula					0.78						0.7	
rh__bankssts					0.71		0.69					
rh__caudalanteriorcingulate				0.73							0.48	
rh__caudalmiddlefrontal					0.76						0.74	
rh__cuneus		0.71						0.72				
rh__entorhinal			0.34						0.34			
rh__fusiform					0.5		0.4					
rh__inferiorparietal	0.31				0.7		0.67					
rh__inferiortemporal	0.31				0.38		0.58					
rh__isthmuscingulate					0.45							
rh__lateraloccipital		0.47					0.37	0.47				
rh__lateralorbitofrontal					0.73						0.72	
rh__lingual		0.66						0.68				
rh__medialorbitofrontal					0.74						0.67	
rh__middletemporal					0.66		0.75					
rh__parahippocampal					0.39							
rh__paracentral					0.83						0.65	
rh__parsopercularis					0.76						0.68	
rh__parsorbitalis					0.62						0.82	
rh__parstriangularis					0.71						0.8	
rh__pericalcarine		0.77						0.82				
rh__postcentral					0.77		0.31				0.35	
rh__posteriorcingulate					0.57						0.44	
rh__precentral					0.96				-0.31		0.83	
rh__precuneus					0.76							0.72
rh__rostralanteriorcingulate				0.44							0.51	
rh__rostralmiddlefrontal					0.7						0.68	
rh__superiorfrontal					0.95						0.81	
rh__superiorparietal					0.82							0.71
rh__superiortemporal					0.91		0.39				0.46	
rh__supramarginal					0.82		0.36					0.43
rh__frontalpole												
rh__temporalpole			0.34									
rh__transversetemporal					0.78					0.39	0.43	
rh__insula					0.73						0.69	
